# Helping patient educators meet health literacy needs: End-user testing and iterative development of an innovative health literacy editing tool

**DOI:** 10.1016/j.pecinn.2023.100162

**Published:** 2023-05-09

**Authors:** Julie Ayre, Danielle M. Muscat, Olivia Mac, Carissa Bonner, Adam G. Dunn, Jason Dalmazzo, Dana Mouwad, Kirsten McCaffery

**Affiliations:** a Sydney Health Literacy Lab, Sydney School of Public Health, Faculty of Medicine and Health, The University of Sydney, NSW, Australia; b Discipline of Biomedical Informatics and Digital Health, School of Medical Sciences, Faculty of Medicine and Health, The University of Sydney, NSW, Australia; c Western Sydney Local Health District, Health Literacy Hub, Westmead, NSW, Australia; d Menzies Centre for Health Policy and Economics, Sydney School of Public Health, Faculty of Medicine and Health, The University of Sydney, Sydney, Australia

**Keywords:** Health Literacy, Health communication, Health information, Tool evaluation, User testing, Readability

## Abstract

**Objective:**

The Sydney Health Literacy Lab (SHeLL) Editor is an online text-editing tool that provides real-time assessment and feedback on written health information (assesses grade reading score, complex language, passive voice). This study aimed to explore how the design could be further enhanced to help health information providers interpret and act on automated feedback.

**Methods:**

The prototype was iteratively refined across four rounds of user-testing with health services staff (*N* = 20). Participants took part in online interviews and a brief follow-up survey using validated usability scales (System Usability Scale, Technology Acceptance Model). After each round, Yardley's (2021) optimisation criteria guided which changes would be implemented.

**Results:**

Participants rated the Editor as having adequate usability (M = 82.8 out of 100, SD = 13.5). Most modifications sought to reduce information overload (e.g. simplifying instructions for new users) or make feedback motivating and actionable (e.g. using frequent incremental feedback to highlight changes to the text altered assessment scores).

**Conclusion:**

terative user-testing was critical to balancing academic values and the practical needs of the Editor's target users. The final version emphasises actionable real-time feedback and not just assessment.

**Innovation:**

The Editor is a new tool that will help health information providers apply health literacy principles to written text.

## Highlights

• Existing health literacy tools do not provide actionable real-time objective feedback.
• The SHeLL Editor provides instant automated feedback on written health information.
• We tested the Editor with health staff to identify how it could be further improved.
• The SHeLL Editor was rated high on usability and acceptability by health staff.
• Key changes were simplifying the user interface and ensuring feedback was actionable.

## 1 Introduction

Health literacy reflects a person's capacity to access, understand, appraise, and use information and services to promote and maintain good health [1]. Systematic reviews show that low health literacy is associated with higher mortality, morbidity, rates of hospitalisation and emergency department visits, and medication errors [2]. However, this is not the full picture. The impact of health literacy on health outcomes is also a reflection of the ‘health environment,’ such as the availability of appropriate health information, services, and other health-related resources.

The clear importance of high-quality, accessible health information is reflected in national and international policies and guidelines [3]. These resources consistently recommend that efforts to address health literacy should ensure that all people can easily access and understand health information. However, this recommendation rarely eventuates in practice. For example, readability assessments repeatedly demonstrate that existing health information is often written 2–4 school reading grades above the level recommended for the general population, and is almost never written at the level recommended for adults with low literacy [[4], [5], [6]]. This clear gap between policy and practice persists, at least in part, because of the absence of effective systems, tools and training that can support health information providers to develop texts that are easy to understand.

Several existing resources provide guidance on how to structure, write, and visually present health information [[7], [8], [9]]. However, there are currently few interactive tools that provide feedback on the health literacy demands of written health information, though see [[10], [11], [12], [13], [14]] for examples. None are widely used. Without such tools, applying health literacy principles to written text can be time-consuming and subjective. There has been some exploratory work into objective, programmed assessments of text complexity that extend beyond school grade reading level [[15], [16], [17], [18], [19], [20]]. However, in health contexts this research has focused on identifying patients with low health literacy or solely on *assessing* text complexity [17,21]. To our knowledge these programmed assessments have not been incorporated into a practical, user-friendly, and free or low-cost tool that gives actionable feedback on individual words or sentences. As such, even though the assessments may be rigorously validated, they are unlikely to provide sufficient feedback to help health information providers (e.g. busy health staff and clinicians) revise and improve existing health texts. To achieve this aim, such tools must also help users interpret the assessments and provide practical feedback about how to simplify the text.

Our team recently developed the Sydney Health Literacy Lab (SHeLL) Health Literacy Editor [22] to address this issue. The Editor is an online tool that objectively assesses the complexity of health materials and provides ongoing feedback in real-time on word choice, and sentence length and structure. This study aimed to explore how the design could be further enhanced to help health professionals interpret and act on automated feedback.

## 2 Methods

### 2.1 Ethical approval

Ethical approval for the study was obtained from Western Sydney Local Health District (project number 2020/ETH02444).

### 2.2 Study design

Iterative user-testing via online interviews that involved think-aloud tasks as participants interacted with the Editor [23]. This type of real-time interview structure combines rich observational and verbal data to explore how users understand, process, and respond to an intervention and its various features [17].

### 2.3 Participants and recruitment

Participants were eligible if they were health staff involved in developing health information materials as part of their role, able to speak, read and write in English, and had access to a computer and internet during the interview. Participants were recruited through seminars and newsletters from the Western Sydney Local Health District's Health Literacy Hub, a locality-based research, development and capacity-building hub comprising more than 1300 health staff, students, and researchers, as well as a regular newsletter about research activities in the District. Interested participants completed an expression of interest form that asked for their demographics and professional information, how often they developed health materials, and how confident they felt applying health literacy principles to written text. Purposive sampling was used to select participants to interview who had diverse experiences, including from a range of disciplines and varying confidence applying health literacy principles. Participants were recruited between 3rd March 2021 and 29th April 2022. Interviews took place online using Skype for Business or Zoom, at the users' preferred location (e.g. work or home).

### 2.4 Intervention

**Fig. 1:**
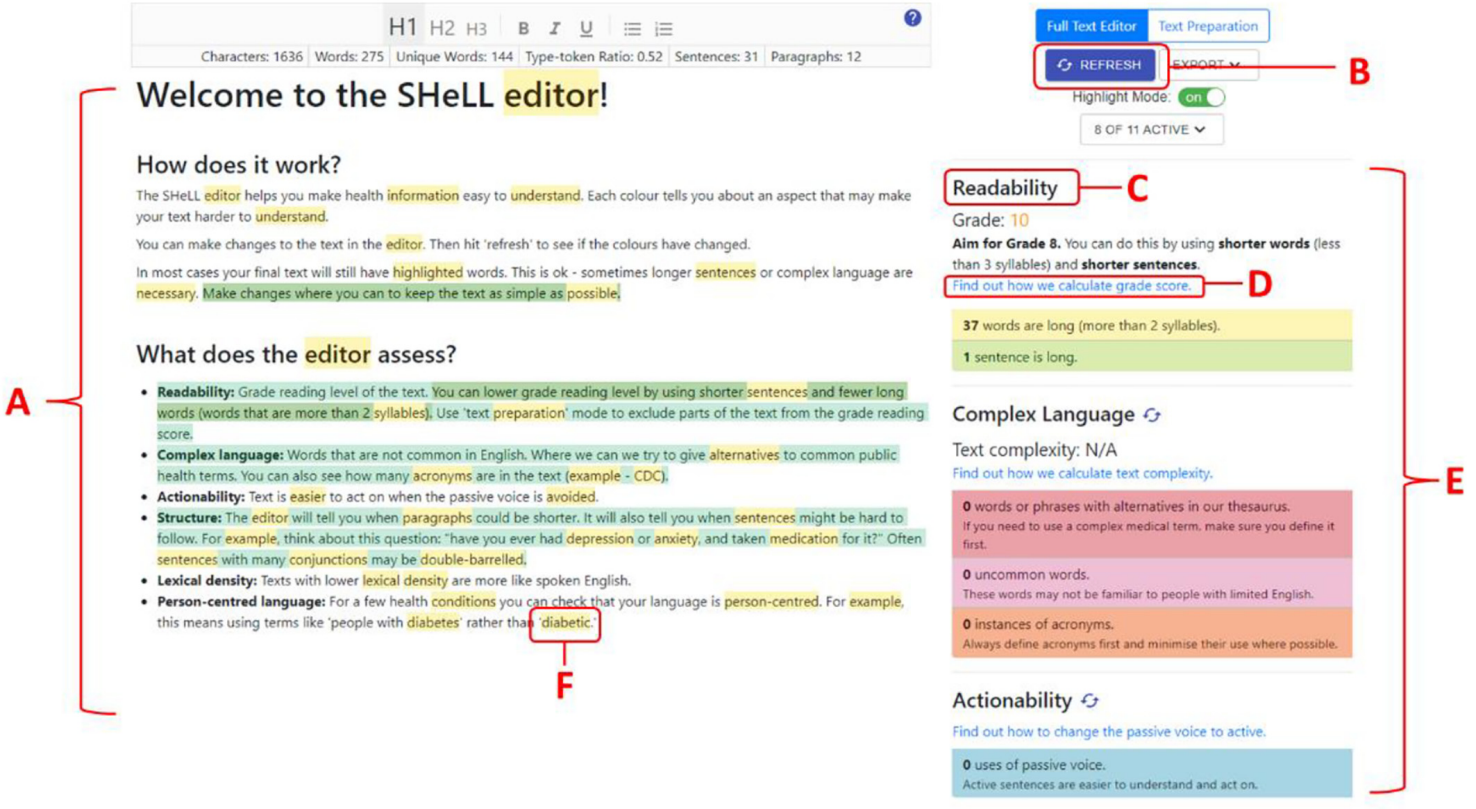
Annotated screenshot of version 1 of the Health Literacy Editor. Notes: A: Left-hand pane for text-editing (shows default text when Editor is first opened); B: Refresh button; C: Example of (global) assessment; D: Link to help page; E: Right-hand pane, shows Assessments; F: Example of text highlight. The colour of the highlight corresponds to a relevant Assessment on the right-hand pane.

The SHeLL Health Literacy Editor is an online browser-based tool that aims to assist health information providers to develop text-based health education materials for patients or community members, that adhere to health literacy guidelines. The Editor comprises six assessments: readability, complex language, passive voice, text structure, lexical density and diversity, and person-centred language. These are each presented as global scores, with additional, more specific feedback flagged in the text itself through coloured highlights (Fig. 1).

When users open the Editor for the first time, they are presented with a brief tutorial to orient them to the most important functions. Further information, tips, and technical notes are accessible via a link to a help page that is embedded within the Editor. A record of the assessments can be downloaded using a printable summary feature.

Greater detail about version 1 of the Editor's development and the rationale for each design decision is provided elsewhere [22]). Broadly, each assessment corresponds to a recommendation in widely-used health literacy guidelines and tools that was amenable to programming (e.g. the Health Literacy Universal Precautions Toolkit [8]). The school grade 8 reading target (equivalent to 13–14 years of age) was based on Australian health literacy guidelines [24,25].

### 2.5 Interviews and user-testing

Participants who took part in the interview were first asked about their experiences of creating written health information and applying health literacy principles (for interview schedule see Appendix A). Participants then engaged in two think-aloud tasks, with methods informed by Willis [23]. Participants were provided a link to the Health Literacy Editor and asked to share their screen. The order of the two think-aloud tasks were alternated across participants:

Task 1: Participants were asked to ‘explore’ the Editor and try out the different features.

Task 2: Participants were provided with an example health text (Appendix B; Grade 12.3), and asked to copy the text into the editor and revise the document based on the Editor's feedback.

During these tasks participants were encouraged to say their thoughts out loud. They received minimal assistance to use the Editor. Neutral prompts reminded participants to continue thinking aloud if they were silent for an extended period of time, and to explore thoughts further. Given the focus on thinking-aloud and exploratory interactions with Editor features, data on time to complete tasks were not recorded, nor were assessments of users' revised texts.

After completing the think-aloud tasks participants were asked for general feedback on the Editor and any support or training that might be helpful. Audio data were transcribed, and videos of the shared screens also recorded. Interviewers (JA and OM) made extensive notes and reflections during and after the interviews.

### 2.6 Analysis and iterative optimisation

Analysis drew from existing methods for using think-aloud studies to optimise digital interventions, which recommend a minimum three to four rounds of user-testing [26,27]. User-testing observations and feedback (positive and negative) were collated. Potential modifications to optimise the intervention were noted. After every five interviews, authors met to discuss which modifications should be incorporated before proceeding with the next round of user-testing. Modifications were prioritised based on the criteria set out by Bradbury, Morton, Band, van Woezik, Grist, McManus, Little and Yardley [26] (Table 1). For the final round of user testing, users had up to 7 days to familiarise themselves with the Editor. This approach provided additional feedback on issues that may arise with longer-term use. Transcripts and observation notes were also analysed thematically to explore overarching and broader concepts relating to users' needs [28]. This involved coding the data, grouping similar codes into preliminary themes, and charting the data into a thematic framework (with each row representing a participant and each column a subtheme or theme). The research team discussed and refined the themes collectively.

**Table 1:** Criteria for deciding when to implement an intervention modification⁎

Criterion	Description
**Criteria for deciding whether to make modifications**	
1. Important for behaviour change	Modification likely to impact behaviour change / precursor to behaviour change (e.g. acceptability, feasibility, persuasiveness, motivation, engagement).
2. Consistent with guiding principles	Modification aligns with guiding principles of the intervention i.e. aspects of the intervention required to achieve its aims (incorporating theory, evidence and user perspectives) Modification aligns with common guiding principles: to support autonomy, promote competence, and provide a positive emotional experience and sense of relatedness
3. Uncontroversial and easy	An uncontroversial and easy-to-implement solution that does not involve major design changes
4. Repeated by several participants	This point was made by more than one participant
**Criteria for prioritising which modifications to make (MoSCoW)**	
5. Must have	Modification must be made for the intervention to be effective in changing a participant's behaviour (given what we know about the evidence base).
6. Should have	Modification should be made if possible because it may impact effectiveness but may be able to be delivered in a different way, or is in some way less critical than a must-have.
7. Could have	Modification would be useful but may be less critical to behaviour change than a should-have and may only be implemented if time and resources are available.
8. Would like	Modification is not needed to support behaviour change but could be useful if time and resources allow.

⁎ Adapted from Bradbury, Morton, Band, van Woezik, Grist, McManus, Little and Yardley [26].

### 2.7 Measures

After the interview, participants completed a brief follow-up survey that included validated questions about usability and acceptability; the System Usability Scale [29,30], and the Technology Acceptance Model (comprises two subscales: perceived usefulness and perceived ease of use) [31]. The System Usability Scale produces a score from 0 (low) to 100 (high). A score of 70 is considered ‘passable,’ and a score of 90 or more is considered indicative of a ‘truly superior product’ [30]. The Technology Acceptance Model subscales produce a score ranging from 1 (low) to 7 (high). Scores are predictive of current and future use of a product [31].

## 3 Results

Out of 51 interested participants, 20 were purposively selected, invited and took part in user-testing. Participant characteristics are shown in Table 2. Participants came from a variety of specialties, including seven (35%) whose role was explicitly about health communication.

**Table 2:** Participant characteristics and usability ratings.

Characteristic	N	%
**Gender**		
Male	2	10
Female	18	90
**Age**		
20–29	6	30
30–39	1	5
40–49	7	35
50–59	6	30
**Role/profession**		
Health communication (role in health literacy, health promotion, or multicultural health)	7	35
Physiotherapy/musculoskeletal	3	15
Speech pathology	2	10
Nurse/midwife	2	10
Podiatrist	2	10
Pharmacist	2	10
Dietician	1	5
Psychologist	1	5
**How often do you develop health education materials?**		
Daily	1	5
Weekly	4	20
Monthly	8	40
A few times a year	7	35
**Usability ratings**	**M**	**SD**
System Usability Scale (0 low to 100 high)	82.8	13.5
Technology Acceptance Model (1 low to 7 high)		
*Perceived usefulness*	5.5	1.1
*Perceived ease of use*	5.8	1.2

Usability and acceptability survey data indicated adequate usability (Mean (M) = 82.8 out of 100, SD = 13.5), perceived usefulness (M = 5.5 out of 7, SD = 1.1) and perceived ease of use (M = 5.8 out of 7, SD = 1.2) (Table 2). These scores were high, even during the first round (M = 90.0, 5.7, and 5.7, respectively). Verbal feedback reflected the high acceptability and usability scores, including during the first round of user-testing. Participants emphasised the value of having feedback in real-time, and for individual words and sentences:

*“I think it is fantastic. Just having a dynamic kind of tool that shows you exactly what the impact is when you make changes to sentences or words or acronyms is great… using this tool, straightaway it's, it gives you a colour coded, guide to, ‘ok, the problem with this is it's too long, the sentence is too long or there's too many syllables’. Or clicking on a word and finding out, ‘oh, ok, I can use a more everyday word’. So I think that the colour coding is fantastic. Um, I think the built in tips are useful as well.”* (Female (F), Health communication).

Further, some participants compared the Editor to other online text-editing software (e.g. Hemingway App, Grammarly), and saw value in having feedback that was specific to health contexts:

*“this is better than [online text-editing tool]. It gives me more information about the text that I'm going to write for these patients, and, it's very specific towards health-based language.*” (F, Podiatrist).

The ability to record the assessments was perceived as valuable at an organisational level:

*“And I liked the reports that you can generate…like if you wanted to get it approved. As a department document you could put that in, and that would be really helpful.*” (F, Speech pathology).

However, there were also many opportunities to refine the Editor further. The following sections detail two key changes to enhance how health information providers can interpret and act on the Editor's feedback: reducing information overload; and making feedback motivating and actionable. A third section details potentially valuable modifications that did not meet the criteria set out in Table 1 (summarised in Table 3).

**Table 3:** Summary of themes for Editor improvement.

Theme / key area for change	Summary
Reducing information overload	• Limit the number of instructions and information presented to new users • Use visual cues where possible to guide new users • Give worked examples for complex concepts
Making feedback motivating and actionable	• Give specific and incremental feedback wherever possible so that users can ‘see’ the relationship between assessments and their revisions to the text • Provide flexibility / customisability where appropriate • Enhance help page to support user action
‘Could have’ and ‘would like’: Potentially valuable (future) features	• Increase automation where possible to reduce user clicks and keystrokes • Increase actionability further e.g. expand thesaurus to include more words • Align with features of other text-editing software (e.g. grammar check) • Develop features that are consistent with broader health literacy principles (e.g. clear key messages)

**Fig. 2:**
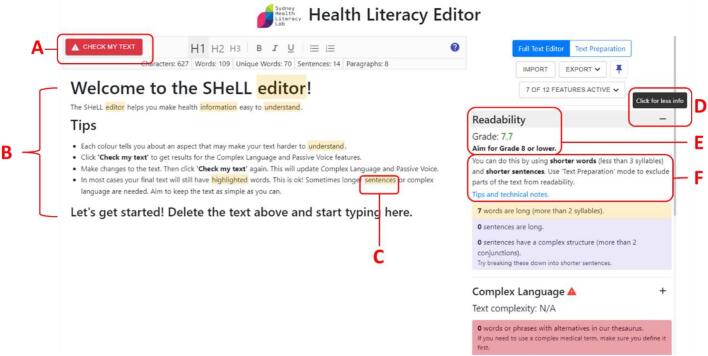
Annotated screenshot of revised Health Literacy Editor. Notes: A: Refresh button (now labelled ‘Check my text’); B: Shorter default text in left-hand pane for text-editing; C: Example of text highlight. The colour of the highlight corresponds to a relevant Assessment on the right-hand pane. When the cursor hovers over a highlight, the colour darkens in the left and right panes, and the right-hand pane will scroll automatically to the corresponding assessment; D: Extra prompt/instructions given when cursor hovers over a feature (cursor not shown); E: Example of default (global) assessment and information; F: Additional information available from collapsible text, includes link to help page.

A full list of modifications is available in Appendix C and screenshot of the final version of the Editor is shown in Fig. 2.

### 3.1 Reducing information overload

During the first round of user-testing, all participants reported that the information and instructions were overwhelming, and that there was too much feedback to take in (including too many coloured text highlights; see Fig. 1) and too many instructions to remember. This was particularly the case for people who had less experience writing in plain language, and those who described themselves as less confident with computers. These initial reactions had a strong bearing on the participants' initial impressions of the Editor and made it harder to learn how to use it effectively. Even before copying the health text (Appendix B) into the Editor, one participant commented on the visual display of the landing screen being complicated (refer to Fig. 1):

*My first thought when I first look at it, it's very busy. Very, very busy. And there's lots of colours [highlighted text]…So now that I've looked at that, it's the exact opposite. So I would have thought green [highlighted text], in my mind green is good.* (F, Dietician).

Modifications to address this issue therefore met criteria 1 (important for behaviour change) and 4 (repeated by several participants) for deciding when to implement an intervention modification. The following changes were also uncontroversial and easy (criteria 3): we simplified instructions, reduced the total number of words in the default text, limited the number of assessments that were turned on by default, and used visual cues to emphasise the connection between text highlights and global assessments. By the final round, participants commented that the initial screen (as shown in Fig. 2) was simple and engaging:

“*but I think that the screen looked clear. It doesn't look scary or over complicated*.” (F, Health communication).

In round 1, participants also asked for more detail about what each of the assessments meant and how they could use the feedback to improve the text:

*“[clinicians] might not really understand what ‘passive voice’ is…some sort of example might help…”* (F, Health communication).

In response we made modifications to layer the information (e.g. providing brief information or examples in the right-hand pane rather than requiring users to access the help page). However, participants in round 2 felt this modification made the right-hand pane too long:

“*so I … got used to looking at like the purple [highlight] for long sentences and then I was like, oh, wait, which one's the pink [highlight correspond to] or, or what does that mean? So you just have to, I had to keep kind of flipping back through [the right-hand pane].*” (F, Outpatient musculoskeletal).

For the remaining two rounds we introduced collapsible text to reduce information overload whilst still providing easily accessible instructions (see Fig. 2, annotation F). After the final round we introduced prompts when users hovered over an assessment (e.g. ‘click for more/less info’ as shown in Fig. 2, annotation D) to make this clearer as some users still had not accessed this function.

Lastly, though the Editor describes critical behaviours in the opening tutorial (e.g. using the ‘refresh’ button), we observed that often participants did not perform these behaviours when editing the text. We introduced prompts elicited by user actions (e.g. user hovers over a related feature) or time elapsed without engaging in the behaviour. By the final round of testing participants reported that these critical behaviours felt more intuitive:

“*It took me a moment to figure out that I needed to [click the refresh button]. But it is very nicely in red and a little symbol comes up on the side as well. So that didn't take too long to get to that.*” (F, Health communication).

### 3.2 Making feedback motivating and actionable

When participants used the editor to revise a text, we noticed that many participants (Table 1 criterion 4) had a strong preference for highly specific advice, for example, thesaurus entries. We also observed that several participants (Table 1 criterion 4) expected to see incremental improvements in overall scores. In the absence of specific and incremental feedback, participants reported feeling uncertain about what action to take, and some ultimately became less engaged in the revision task.

Modifications to ensure that feedback was both motivating and actionable were considered inherently important for behaviour change (Table 1 criterion 1) and consistent with the Editor's guiding principles (Table 1 criterion 2). These modifications are described in detail below.

1. **Grade reading score to 1 decimal place:** In the original version of the Editor, grade reading score was presented without a decimal place. This was intended to reflect the fact that small differences of less than 1 grade score are not meaningful. However, we observed that participants often checked whether relevant changes (e.g. using shorter words and sentences) were making any difference to the grade reading score. By providing the grade reading score to 1 decimal place participants could more easily see the text improving incrementally. This was demonstrated by the following participant, who continually referred to the grade reading score as they made small edits to the text:
  *“Oh, so now the readability is still 9.5. Yeah, ok. [edits the first sentence of the example text Appendix B)]…so [now] I'm at 9.1”.*
  For the above change in readability, the participant broke a long sentence (purple highlight) into two and changed two long words (yellow highlights). The participant edited the original sentence ‘*Preventing cardiovascular* disease (CVD) means making smart choices now that will pay off the rest of your life’ to read ‘To stop heart disease from happening we need to make smart choices. That will pay off the rest of your life.’
  The participant then tried editing the sentence ‘Everyone can benefit from a healthy diet and adequate physical activity.’ As shown in the quote below, they continued to check for incremental improvements after making small changes:
  *…Ok, let's, let's see what words we can remove. Maybe ‘adequate’. Let's say ‘enough’ [instead]. Enough… does that change grade reading score? Oh, we are now 9. That's not bad (laughs).”*
  (F, Mental health pharmacist)
2. **Additional actionable readability highlights:** The ‘complex sentence’ assessment was moved into the ‘readability’ feature as the accompanying advice was relevant to readability i.e. breaking a sentence into two. An additional readability assessment was created to alert users of long lists within a sentence. This change meant that some long sentences would have more actionable feedback (i.e. from ‘X sentences are long’ to: ‘try breaking [the sentence] down into shorter sentences’ and ‘use dot points for lists of more than 4 things’).
3. **Flexibility in counting long words and complex language:** Participants sometimes expressed frustration when long words (readability) or words with a thesaurus entry (complex language) were one of the key words for the text. We modified the Editor to allow up to 5 words to be excluded from complex language assessment, including words listed in the thesaurus. We also modified the printable summary to show the impact of these words on grade reading score. This supplementary assessment provides users with more nuanced and pragmatic ways of demonstrating that a text has followed health literacy principles. For example, if users are creating a text about diabetes, it may be challenging to obtain low complex language or readability scores because the topic word (‘diabetes’) is considered complex, more than 2 syllables, and is likely to be repeated several times. With this modification, users can entirely exclude the term from complex language counts. They can also observe the grade reading score with and without the word ‘diabetes.’ This pragmatic approach allows users to focus their efforts on long words that are more appropriate to revise and seeks to limit frustration with the tool. Note that excluding words from grade reading scores is not standard practice, and for this reason the ‘revised’ grade reading score is only shown in the printable summary.
4. **Pinned results:** An additional feature was created to allow the user to ‘pin’ assessments of the original text. This allows the user to easily compare results and emphasises incremental improvements as they continue to make revisions: “*Yeah, like how you can pin a previous result. That was good.”* (F, Speech pathology)
5. **More actionable help page:** Initially the help page contained mostly technical notes about how scores were calculated and the underlying resources. Feedback from users indicated a need for tutorial videos, brief instructions for using the Editor, and worked examples of how text could be revised. These sections were added and technical notes were hidden in sections of collapsed text. By the final round participants commented that the help page was useful:
  *“I found the videos great…They were really clear, concise, they weren't too lengthy. The audio … came across as knowledgeable but approachable. Like it was engaging. So, yeah, they were really helpful.”* (F, Health communication).

### 3.3 ‘Could have’ and ‘would like’: potentially valuable (future) features

This section highlights modifications that met criteria for being important for behaviour change, being consistent with guiding principles, and being raised by several participants (Table 1, Criteria 1, 2 and 4, respectively). However, we were unable to implement these changes due to limited time and resources. Though almost all participants expressed that the thesaurus function was very valuable, requests to further enhance the thesaurus feature were also common. For example, the following participant discussed wanting a more extensive medical thesaurus for words such as some medical jargon is flagged as uncommon but no suggested alternatives are provided:

*“While hovering over the word ‘fissure’] So I'm wondering whether or not it would be a really great addition if you could … right click [on] the pink highlighted [uncommon] words that would give you a different option.”* (F, Podiatrist).

Others went even further, suggesting a simple English thesaurus for all uncommon words, rather than one focused on medical and public health jargon.

Many also expressed interest in being able to click on a thesaurus suggestion to replace the highlighted word in the text:

*“I think … it would have been cool if you could've like clicked on one of the suggestions and it just auto-populated it for you, rather than you have to type it in yourself*.” (F, Outpatient musculoskeletal).

Both modifications require substantial programming and further user testing, and as such were outside the scope of the current project. In lieu of these changes we modified the Editor to explain these limitations more clearly, e.g. “Where we can, we try to give alternatives to common public health terms”.

Other potentially valuable features included grammar checks.

*“But just grammar and a spell check, ‘cause I think the way a lot of our staff write at the moment, they assume there's a spell check and a grammar check.*” (F, Physiotherapist).

Again, this modification was not feasible within the constraints of the project resources.

Lastly, one participant described the potential undesirable consequences of focusing on simplifying the language at the expense of broader considerations about the key message and tone:

“*I wonder… is that core objective of the piece of writing missed because you've plain-languaged it, you've changed it all around, and then the shift or the tone changes…yeah, like something could be plain-languaged, … and ticks all the boxes, but is it doing what it's meant to do?*” (F, Health promotion).

Again, additional features to keep users focused on key messages could be incorporated into future iterations of the Editor. In response to this comment we incorporated guidance about retaining key messages into the help page.

## 4 Discussion and conclusion

### 4.1 Discussion

This study reports systematic user-testing and iterative improvement of an innovative health literacy text-editing tool, the SHeLL Health Literacy Editor. Overall participants reported that they valued the Editor's ability to give real-time, specific feedback on written text, including aspects specific to health contexts. Over four rounds of testing we refined the tool's instructions, layout, and visual feedback, and introduced some new features. Most of these modifications sought to address two issues: 1) reducing information overload for new users; or 2) providing feedback on the user's text that is actionable and motivating. The second of these issues was addressed by making feedback more specific where possible, and emphasising the user's incremental improvements to the text. Features to enhance the thesaurus feature and broaden its scope were very popular but not feasible for the current project.

Reducing information overload is critical for learning new tasks. Learning how to use the Editor for the first time is no exception. New users must quickly learn how to operate the Editor's basic functions (e.g. remembering to press the ‘refresh’ button), what each assessment means, and interpret the assessment feedback. Modifications to better achieve these learning goals aligned with established strategies to reduce cognitive load, such as reducing initial complexity, reducing requirements for split attention, using worked examples, and providing ‘just-in-time’ prompts to encourage correct use of basic functions [[32], [33], [34]].

Providing feedback was a central theme in this study, and is a common component for educational or training interventions for healthcare providers. Theoretical models from behavioural and education sciences emphasise that feedback can be enhanced by identifying specific targets and action plans to help users meet these targets [[35], [36], [37]]. Theories also advocate feedback that is timely, individualised, and non-punitive, and can be customised to individual needs [[38], [39], [40]]. Findings from this study align with these models. From the start, participants appreciated the real-time feedback on specific words and sentences. Modifications further enhanced the clarity, specificity, and timeliness of feedback, and enhanced flexibility in how readability and complex language were calculated.

This study also highlighted aspects of writing in plain language that the Editor does not provide feedback on. For example, a few participants were interested in grammar checks, particularly those who spoke English as a second language. One participant emphasised that it can be hard to simplify a text if the key messages are not clear. In its current form, the Editor does not help users identify their key messages nor identify extraneous information. Additional training resources such as videos may go some way to address this limitation. Ultimately, though, we advise that health information providers use the tool in conjunction with other existing health literacy guidelines and resources [[7], [8], [9]], and importantly, gather feedback from their target users i.e. patients and community members, as the latter has shown to significantly improve health information materials [41].

The strengths of this study were that we employed a systematic approach to user-testing. As a result, participants matched our target users. Participants were health staff from a range of ages, different specialities, and varying experience writing in plain language, although few men took part. We also used systematic methods to reflect on and prioritise potential modifications between each round of testing [27]. The key limitation was that some participants may have felt pressure to perform when they were observed and asked to think aloud while revising the practice text. The final cycle allowed participants to familiarise themselves with the Editor first. This may have addressed the issue to some extent, though it is likely that some participants would engage with the Editor differently if they were not observed. Lastly, this study focused on how users interact with the Editor. Several avenues of future work are required for more comprehensive evaluation. First, the Editor's feedback should be compared to other existing tools to assess their accuracy and scope. For example, we have demonstrated that the Editor provides more accurate SMOG grade reading scores than other widely used readability calculators [42]. The same process could be applied to the Editor's other assessments. Second, we need to evaluate how effectively the Editor supports health information providers to write in plain language. Third, we need to evaluate how it can best be implemented into health organisations.

### 4.2 Innovation

This study provided a useful case study demonstrating how an innovative intervention can be further enhanced through a rigorous process of iterative user-testing. The SHeLL Health Literacy Editor is an interactive tool to support health information providers learn about health literacy principles, and then apply these principles to written text in real-time. Formal user-testing was critical to adjusting the balance between academic values (e.g. comprehensiveness, transparency, and accurate assessment), and the practical needs of the tool's target users. The modifications reduced information overload and improved the capacity for feedback to support effective revision of the text. User-testing is an area that is often overlooked or not publicly documented when health literacy tools are developed. This happens despite the recognised importance of user-testing in sustainable and impactful implementation [27].

The Editor may offer other indirect benefits. For example, it may support improvements to the quality of translated health information. This is because English-language parent texts that are written more simply are also more accurately translated [43]. To illustrate, the Editor has been used to develop a simple-English glossary of COVID-19 vaccine terms that is now translated into 29 languages [44]. More broadly, this study contributes to our understanding of health information providers' experiences of applying health literacy principles to written text, and may be useful in developing other health literacy training interventions and resources.

### 4.3 Conclusion

The SHeLL Health Literacy Editor is a text-editing tool that moves beyond *assessment* of text complexity to provide timely and actionable feedback to help health information providers apply health literacy principles to written text. Rigorous user-testing with health information providers provided rich data to make the Editor more intuitive, appealing, and engaging.

## Funding

Development of the Editor was supported by a Lifespan Research Network seed funding grant at the 10.13039/501100001774University of Sydney. This user testing study did not receive any specific grant from funding agencies in the public, commercial, or not-for-profit sectors. In-kind support for this study was provided by the Health Literacy Hub (10.13039/100014467Western Sydney Local Health District).

## Declaration of Competing Interest

The authors declare that they have no known competing financial interests or personal relationships that could have appeared to influence the work reported in this paper.

The authors declare the following financial interests/personal relationships which may be considered as potential competing interests:

This study was primarily funded via the Lifespan Research Network seed funding. Development of the Editor was supported by a Lifespan Research Network seed funding grant at the 10.13039/501100001774University of Sydney. This user testing study did not receive any specific grant from funding agencies in the public, commercial, or not-for-profit sectors. Dana Mouwad reports administrative support was provided by Western Sydney Local Health district, Health Literacy Hub. Administrative support was provided by Western Sydney Local Health Distrcit, Health Literacy Hub. Academic authors were supported by National Health and Medical Research Council, Heart Foundation, and Western Sydney Local Health District. Members of the research team (JA, DMM, CB, KM) are directors of a health literacy consultancy (Health Literacy Solutions Ltd., Pty).

## Appendix A Supplementary data

Supplementary material Supplementary material includes (A) interview guide; (B) Example health text; and (C) List of all key modifications Supplementary material
