# Supplementary material for: Helping patient educators meet health literacy needs: End-user testing and iterative development of an innovative health literacy editing tool
Source: PEC Innov. 2023 May 9;2:100162. doi: 10.1016/j.pecinn.2023.100162 (PMC10294045; doi:10.1016/j.pecinn.2023.100162)
Supplement: Supplementary material — includes (A) interview guide; (B) Example health text; and (C) List of all key modifications [file mmc1.docx]

# Appendix A: Interview Guide

**Part 1. Understanding current health literacy strategies in use**

1. Can you tell us a bit about your process for developing health information written for patients or people in the community? *[Prompt: what are the main strategies you use? What kinds of things do you think about when you try to make the language simpler? Easier to act on?]*
2. What do you find most challenging about this process? Easiest? *[Prompt to ask what is most challenging in terms of revising the text]*
3. What resources do you find helpful? *[Prompt: any tools or guides that they use]*

**Part 2. Editor use and feedback**

***Introduction***

In the next part of the interview, what we’d like you to do is ‘think aloud’ about what you are doing as you look at the editor, so we can understand the process you went through and what you thought about it. To help you get used to thinking aloud, we will do a practice exercise first, which is not related to the editor. Then I will show you the editor prototype and give you a document to edit. I won’t be able to answer any questions about the actual task, just do whatever you think is best, and make sure you continue to think aloud the entire time. I will prompt you to keep talking if you are silent for more than 10 seconds. I will also ask you some questions at the end. Does that sound ok? Do you have any questions?

***Practice Task***

Ok, now we will do the practice task. Try to visualize the place where you live, and think about how many windows there are in that place. As you count up the windows, tell me what you are seeing and thinking about.

*[Prompt to keep talking, and give feedback on thinking aloud frequency at the end. Other probes in the think aloud tasks below may include* *“What are you thinking now?”, “What do you think that means?”, “What do you think that’s there to do?”, “What do you expect to see next?” “Was this what you expected?”, “How do you feel about that?”, “How did you come up with that text revision?”, “What made you make that revision?” ]*

***Editor prototype***

Now I will show you the health literacy editor [send link through chat]. Please open the editor and the word document from our previous email. Please share your screen so that I can see the editor in your browser.

[Order of tasks 1 and 2 below alternated across participants]

Task 1: Now your task is to explore the editor. Take your time to play around with what happens when you edit text. You can edit the text in any way you like, and you don’t have to use all the features in the editor. You can copy the practice text into the editor if you like. Please remember to think aloud as you go. I won’t be able to answer any questions about the actual task, just do whatever you think is best. *[Allow participant to play with the editor, thinking aloud as they go.]*

Task 2: Please copy and paste all of the text from the word document into the editor. [wait for this to be done, provide assistance if needed]. Thank you. Now I’d like you to try revising the document using feedback from the editor. If you need to, you can copy the original text in again. Please remember to think aloud about what you see and the edits that you make as you go. I won’t be able to answer any questions about the actual task, just do whatever you think is best.

Let me know when you finish, and then I will ask you some questions. [When they have finished, ask participant to copy the final version of the text into a word document to email to the researcher]

Final editor prototype questions

1. What did you think of this editor? *[Prompt: what did you like or dislike about it? Was anything unexpected? Clunky?]*
2. The editor has several features. Which ones do you think worked well? Which didn’t work well or weren’t useful? Why?
3. What would you have liked to have seen in this editor?
4. What kind of training/support do you think would be useful and acceptable? [*Prompt for different formats e.g. recorded seminars, videos etc]*
5. Any other comments?

# Appendix B: Example health text

Preventing cardiovascular disease (CVD) means making smart choices now that will pay off the rest of your life.

Lack of exercise, a poor diet and other unhealthy habits can take their toll over the years. No matter what your age, everyone can benefit from a healthy diet and adequate physical activity.

Choose a healthy eating plan. The food you eat can decrease your risk of CVD.

Choose foods low in saturated fat, trans fat, and sodium. As part of a healthy diet, eat plenty of fruits and vegetables, fiber-rich whole grains, fish (preferably oily fish - at least twice per week), nuts, legumes and seeds and try eating some meals without meat. Select lower fat dairy products and poultry (skinless). Limit sugar-sweetened beverages and red meat. If you choose to eat meat, select the leanest cuts available.

Be physically active. You can slowly work up to at least 2½ hours (150 minutes) of moderate-intensity aerobic physical activity (e.g., brisk walking) every week or 1 hour and 15 minutes (75 minutes) of vigorous intensity aerobic physical activity (e.g., jogging, running) or a combination of both every week.

Additionally, on 2 or more days a week it is recommended that you do muscle-strengthening activities that work all major muscle groups (legs, hips, back, abdomen, chest shoulders, and arms). Children should get at least 60 minutes of activity every day.

It's never too early or too late to learn the warning signs of a heart attack and stroke. Not everyone experiences sudden numbness with a stroke or severe chest pain with a heart attack. And heart attack symptoms in women can be different than men.

# Appendix C: List of all key modifications

**Key modifications for each round of user-testing**

| **Round** | **Issue (theme)** | **Examples of modifications to resolve issue** | **Example quote / observation** |
| --- | --- | --- | --- |
| Round 1 (n=5) | Tutorial perceived as wordy and unclear | - Signposted each tutorial step using headings - Tutorial introduces the refresh button *after* complex language feature explained. | I can’t remember if [the tutorial] had a clear title on that? Like, … ‘click through these to have a play’ or something. So that it’s just clearer that that’s not part of the analysis itself. (04) |
|  | First use was overwhelming because of the number of colours and amount of feedback | - Changed highlight colours to avoid key colours being green, yellow and orange for different features (these were interpreted as good/ok/poor) - Only 3 assessments turned on by default (Readability, Complex Language, Passive voice) | My first thought when I first look at it, it’s very busy. Very, very busy. And there’s lots of colours…So now that I’ve looked at that, it’s the exact opposite. So I would have thought green, in my mind green is good. (01) |
|  | Users were not hitting ‘refresh my text’ button | - Changed button colour to red, with text that reads ‘Assess my text’ and more prominent placement | maybe like a box on the [top or the] left hand side that was flashing, and maybe you could say ‘click refresh’, ‘click this button to refresh the scores’, or something like that? (03) |
|  | Users wanted quick information about each assessment | - Text under each assessment summary revised to provide more information. E.g:   - Readability: Aim for Grade 8 ‘*or lower*’ added to emphasise aiming for lower scores   - Passive voice: examples provided. | also explaining what grade means, because people might think it’s eight out of ten or something…… rather than year 8 at school. (03)  [clinicians] might not really understand what ‘passive voice’ is…some sort of example might help...(03) |
| Round 2 (n=5) | Users were not hitting ‘refresh my text’ button | - ‘Assess my text’ button text changed to ‘Check my text.’ - The button ‘shimmers’ until pressed, and two minutes later, another shimmer prompts users. This feature is only present to new users (i.e. no cache). | Observed that participants were not often clicking the refresh button. |
|  | Too much text in the right-hand pane | - Explanatory text on right-hand pane made collapsible | what would be really cool is, would be if [sections in the right hand pane] were collapsed and so … if I knew what the headings were… So if there was a little, um… marker that, that [feature] needed attention and then I could just open that particular one (06) |
|  | Actionability assessment not intuitive (other) | - Changed ‘actionability’ to ‘Passive Voice’ | I wouldn’t even have associated the heading ‘actionability’ with the use of passive or active voice. (06) |
|  | Difficulty knowing how to interpret different highlight colours | - When the cursor hovers over a highlight, the right-hand pane will automatically scroll to make the corresponding assessment visible to users. | you had to kind of keep going like in checking what, which colour meant what. Um, so I was like, you know, got used to looking at like the purple for long sentences and then I was like, oh, wait, which ones the pink or, or what does that mean? So you just have to, I had to keep kind of flipping back through. (08)  it would have been good if you could like highlight over it and it came up with what the problem was.(08) |
| Round 3 (n=5) | Users wanted to better see how they were incrementally improving readability | - Presenting grade reading score to 1 decimal place more clearly shows incremental improvements to the text. - ‘Pinned results’ allows users to store results for an ‘original’ version of the text | Observed frustration that the grade reading score didn’t change in response to text edits  Yeah, I think that would be helpful. A clean version and a revised version. (14)  I would like to see like a revised copy and a non-revised copy.(14) |
|  | Users felt that sometimes long words were inherent to the text and meant a Grade 8 reading score was not feasible | - Printable summary shows impact of up to 5 words on grade reading score. This means that the overall grade reading score remains the same, but users can gain a sense of how the topic/condition/illness may have contributed to a higher score. | Observed some frustration with long words e.g. ‘physical activity’ (both count as long words) |
|  | Text preparation pane and other advanced features not used or considered difficult to grasp | - Added short video tutorials (overview, readability, complex language, passive voice), and linked to this in the orientation (initial) tutorial. - Revised text and headings in text preparation pane to makes its functions clearer. | I’m still not clear on what the difference is between those two [modes]… I can see that I can’t, I can’t change it in text preparation but I’m not clear on why I need that. (15) |
|  | More instruction about what to do with long sentences could be helpful | Complex sentence moved to readability as the instruction to break down the sentence is relevant to lowering grade reading score. Added a feature that identifies potential lists that could be turned into dot points. | Observed that users may need more support to revise long sentences.  … like one of those phrases or sentences that I really struggled to make shorter, maybe some suggestions about like formatting it as bullet points or formatting it in a particular way to make it, er, to make the sentence shorter and kind of more readable. Um, that might be helpful. (13) |
|  | Users do not tend to use collapsible panels | Changed ‘click for more’ collapsible panel format | Observed that this was not frequently clicked by users |
| Round 4 (n=5) | Users do not tend to use collapsible panels | - Added a tooltip (box appears on cursor hover) to explain ‘click for more info’ / ‘click for less info’ | Observed that this was not frequently clicked by users |
|  | Users sometimes forgot to click ‘check my text’ after the first few times | - An additional prompt appears once 5 minutes has elapsed since the last time the button was pressed. This can be turned off. | Observed.  I think the only other thing that I needed a reminder on (laughs) was to press I think it was a red button, to …Check my text. Yes, and I, I didn’t do that. (17) |
|  | Some thesaurus entries were not relevant, but thesaurus entries could not be excluded from complex language | - Thesaurus entries can be excluded from complex language counts. The thesaurus entry is still accessible but the highlight is whited-out. | Observed. |
